# Fabrication of double core–shell Si-based anode materials with nanostructure for lithium-ion battery†

**DOI:** 10.1039/c7ra13606d

**Published:** 2018-03-01

**Authors:** Pengfei Wu, Changqing Guo, Jiangtao Han, Kairui Yu, Xichao Dong, Guanghui Yue, Huijuan Yue, Yan Guan, Anhua Liu

**Affiliations:** College of Materials, Key Laboratory of High Performance Ceramic Fibers, Xiamen University Xiamen 361005 China ahliu@xmu.edu.cn; College of Materials, Fujian Key Laboratory of Advanced Materials, Xiamen University Xiamen 361005 China; College of Materials, Department of Materials Science & Engineering, Xiamen University Xiamen 361005 China; College of Chemistry, State Key Laboratory of Inorganic Synthesis & Preparation Chemistry, Jilin University Changchun 130012 China; College of Chemistry and Molecular Engineering, Peking University Beijing 100871 China

## Abstract

Yolk–shell structure is considered to be a well-designed structure of silicon-based anode. However, there is only one point (point-to-point contact) in the contact region between the silicon core and the shell in this structure, which severely limits the ion transport ability of the electrode. In order to solve this problem, it is important that the core and shell of the core–shell structure are closely linked (face-to-face contact), which ensures good ion diffusion ability. Herein, a double core–shell nanostructure (Si@C@SiO_2_) was designed for the first time to improve the cycling performance of the electrode by utilising the unique advantages of the SiO_2_ layer and the closely contacted carbon layer. The improved cycling performance was evidenced by comparing the cycling properties of similar yolk–shell structures (Si@void@SiO_2_) with equal size of the intermediate shell. Based on the comparison and analysis of the experimental data, Si@C@SiO_2_ had more stable cycling performance and exceeded that of Si@void@SiO_2_ after the 276^th^ cycle. More interestingly, the electron/ion transport ability of electrode was further improved by combination of Si@C@SiO_2_ with reduced graphene oxide (RGO). Clearly, at a current density of 500 mA g^−1^, the reversible capacity was 753.8 mA h g^−1^ after 500 cycles, which was 91% of the specific capacity of the first cycle at this current density.

## Abstract 2

Double core shell structure can not only improve the efficiency of lithium transport, but also effectively alleviate volume expansion.

## Graphical abstract

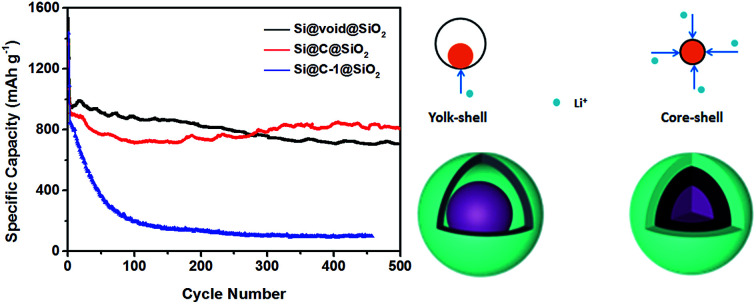

## 1 Introduction

Lithium-ion batteries play an important role in the energy storage because of their long life, light weight and environment friendliness. The development of lithium-ion batteries has also promoted continuous breakthroughs for applications in electric vehicles and digital products.^1,2^ It is worth mentioning that graphite electrode, although widely used in commercial batteries, cannot meet the industrial demand because of its unsatisfactory specific capacity (∼372 mA h g^−1^), indicting the importance of a more remarkable energy intensive alternative.^3^

Previous studies revealed that alloy-type anode materials, such as germanium, silver, and silicon, possess higher specific capacity.^4–10^ Interestingly, the theoretical specific capacity of silicon could reach 4200 mA h g^−1^,^11^ and it is considered to be a strong competitor of the next generation of lithium ion battery anode materials^12^ due to its lower discharge potential (<0.5 V *vs.* Li/Li^+^)^13^ and abundance in the earth's crust. However, this material encounters several challenges in practical applications. (1) A large volume change (∼400%) occurs during the process of discharging and charging, which leads to structure pulverization, loss of electrical contact and decrease in cycling stability. (2) The drastic volume expansion/contraction of silicon during cycling results in an unstable solid electrolyte interface (SEI) layer on the surface of silicon particles, which excessively depletes electrolyte or lithium ions and decreases the specific capacity.^11,14,15^ (3) The conductivity of silicon is poor. In short, these problems severely hinder the commercial development of silicon electrodes.

In the past few years, it has been found that nanocrystallization can solve the grinding problem of silicon.^16–18^ The study of silicon nanomaterials with different shapes has greatly promoted the development of silicon-based electrodes in the form of nanowires,^19^ nanotubes,^20^ and nanoparticles.^21^ Furthermore, various conductive materials comprising silicon have been proven to improve the conductivity of silicon-based electrodes.^22–26^ However, the direct contact between silicon and electrolyte still leads to the consumption of a large amount of electrolyte to form a thick SEI layer, leading to the decrease in specific capacity. Hence, it is important to coat silicon-based materials to avoid contact with electrolyte.

In order to avoid direct contact between silicon and electrolyte, two structures are most commonly used. (1) Core–shell structure, which could protect silicon by coating one or more layers of materials on the silicon surface.^27^ The disadvantage of this structure lies in the silicon leakage through the binding of the shell and further exposure to electrolyte upon volume expansion. However, in the study of silicon core–carbon shell materials by Luo *et al.*, the self-elasticity of the carbon layer can compensate for silicon expansion by adjusting the thickness of carbon layer to a certain level;^28^ (2) the study of yolk–shell structure provides new ideas for applications in various fields, including electrode materials.^29^ For example, Cui *et al.* designed a carbon shell for yolk–shell structure,^22^ while Yang *et al.* successfully synthesized double yolk–shell structure with carbon and silica as shell materials by selective chemical etching.^30^ Furthermore, our group successfully designed yolk–shell structure of other electrode materials in our previous study.^31^

In this study, an anode with sandwich-like double core–shell was designed, in which the outer SiO_2_ layer was the robust shell and the C layer between the shell and silicon core was the buffer layer. In this double core–shell structure, the silica layer and carbon layer can avoid the direct contact between silicon spheres and electrolyte, thus ensuring a thin and stable SEI layer outside the silica shell to avert redundant irreversible reactions during cycling. More interestingly, this structure could avoid the wastage of sacrificial layer and the otiose production of toxic substances when preparing yolk–shell. To the best of our knowledge, there are only few reports on the influence of ion transport ability of these two structures on cycling performance. This study shows that, the discharge of double core–shell structure arises at about the 150^th^ cycle and overtake the performance of yolk–shell structure after the 270^th^ cycle persistently, although its initial discharge capacity is poorer followed with a downtrend. In addition, it is worth mentioning that the conductivity and electrochemical performance improved by compositing with reduced graphene oxide (RGO).

## 2 Materials preparation

### 2.1 Synthesis of Si@RF particles

First, 0.3 g silicon nanoparticles (average diameter of ∼100 nm) were dispersed in a mixture of 180 mL deionized water and 0.92 g CTAB by sonication for 30 min. Then, 59 mL ethanol, 0.4 g resorcinol and 0.1 mL concentrated ammonium hydroxide (28 wt%) were added to the above solution and stirred at 30 °C for 30 min. Further, 0.4 mL formaldehyde was injected with a syringe into the mixture and then, the mixture was stirred for another 16 h. The product, *viz.*, Si@RF particles were collected by centrifugation and washed with water and ethanol several times and then dried in a vacuum oven at 30 °C overnight. The Si@RF particles were thus obtained. The amount of resorcinol was changed to 0.28 g and the resultant spheres were named Si@RF-1.

### 2.2 Synthesis of Si@RF@SiO_2_ particles

Initially, 0.3 g Si@RF core–shell spheres were homogeneously dispersed in 15 mL ethanol, 2 mL deionized water and 0.6 mL concentrated ammonium hydroxide by sonication for 1.5 h. Subsequently, 0.1 mL tetraethyl orthosilicate (TEOS) under stirring at 500 rpm at 30 °C was injected into the system every 0.5 h for three times. The Si@RF@SiO_2_ particles were collected by centrifugation and washed several times with water and ethanol.

### 2.3 Synthesis of Si@C@SiO_2_ and Si@void@SiO_2_ particles

The Si@RF@SiO_2_ sandwich-like spheres were carbonized under N_2_ atmosphere at 350 °C for 2 h, followed by a further heat treatment at 800 °C for 4 h. Both of these heating processes were performed at a heating rate of 1.5 °C min^−1^. Finally, Si@C@SiO_2_ spheres were obtained. The remaining Si@RF@SiO_2_ sandwich-like spheres were incinerated at 500 °C for 1 h in a muffle furnace. Then, the RF layer was cleared away and a yolk–shell structure named Si@void@SiO_2_ was obtained. Si@RF-1 was transformed into Si@C-1@SiO_2_ by the same method.

### 2.4 Synthesis of Si@C@SiO_2_/RGO composites

Initially, 0.12 g Si@C@SiO_2_ spheres and 0.06 g graphene oxide were separately dispersed in 60 mL deionized water by sonication. Then, graphene oxide dispersion was poured into the Si@C@SiO_2_ dispersion, followed by sonication for 2 h. Next, deionized water was evaporated at 65 °C under mechanical stirring. The residue was heated in a quartz boat filled with H_2_/Ar gas mixture and the temperature was increased to 800 °C at the rate of 10 °C min^−1^. The resultant sample was named Si@C@SiO_2_/RGO.

### 2.5 Characterizations

The sandwich-like structures and diameters were characterised on a JEM-2100 (Japan) transmission electron microscope (TEM). The morphologies of these particles were further investigated using a scanning electron microscope (SEM, Hitachi SU-70, Japan). The thermal decomposition behaviour was characterized using a NETZSCH TGA/STA 409 EP analyser in the temperature range from room temperature to 900 °C in air with a heating rate of 5 °C min^−1^. The crystal structure and phase of the products were characterised by a wide-angle X-ray diffraction (XRD, Bruker-axs, Germany) with Ni-filtered Cu Kα radiation (40 kV, 40 mA). Raman spectra were collected using HORIBA with a CCD detector; the wavelength used was 532 nm and the grating was 1800 lines per mm.

### 2.6 Electrochemical characterizations

The as-synthesized particles were mixed with carbon and polyvinylidene fluoride (PVDF) in weight proportion of 7 : 2 : 1. Then, *N*-methyl-2-pyrrolidone (NMP) was added to obtain uniform slurry. The slurry was smeared on a copper foil by a glass rod and then dried at 120 °C in a vacuum oven. CR2032 coin cells were fabricated using the slurry-smeared copper foil as the working electrode, lithium metal chips as the counter/reference electrode and 1 M of LiPF_6_ in a 151 (v/v) mixture of ethylene carbonate (EC) and diethyl carbonate (DEC) as the electrolyte. Celgard 2400 membrane was used as the separator. The cells were assembled in an argon-filled glovebox. The cells were tested between 0.01 and 3.0 V with the Neware battery test system. The EIS curves were obtained by applying a sine wave at a frequency range of 100 kHz to 10 mHz to the assembled cells before cycling.

## 3 Results and discussion

**Fig. 1:**
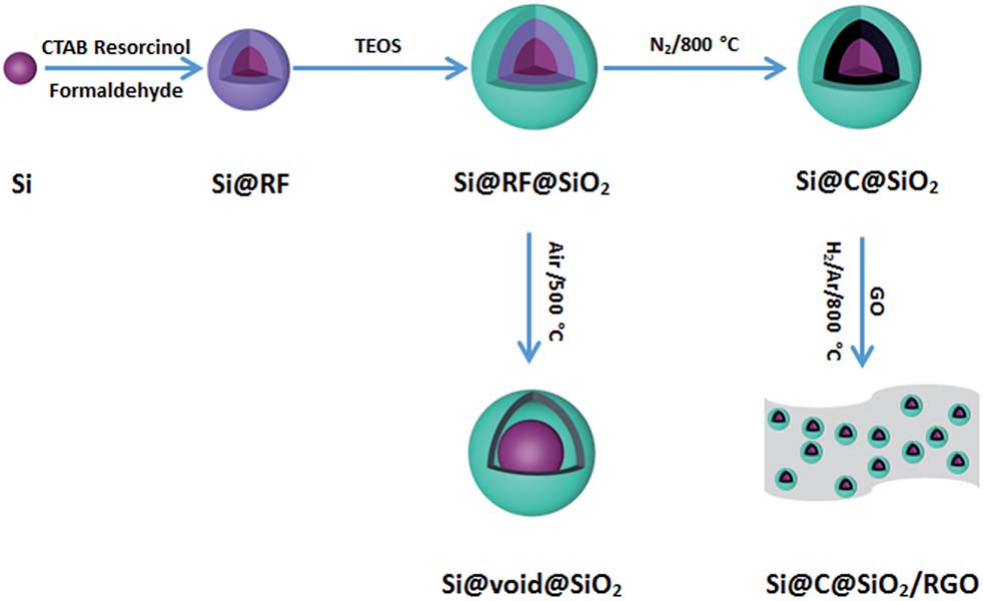
Schematic illustration of the fabrication process for Si@void@SiO_2_, Si@C@SiO_2_ and Si@C@SiO_2_/RGO.

**Fig. 2:**
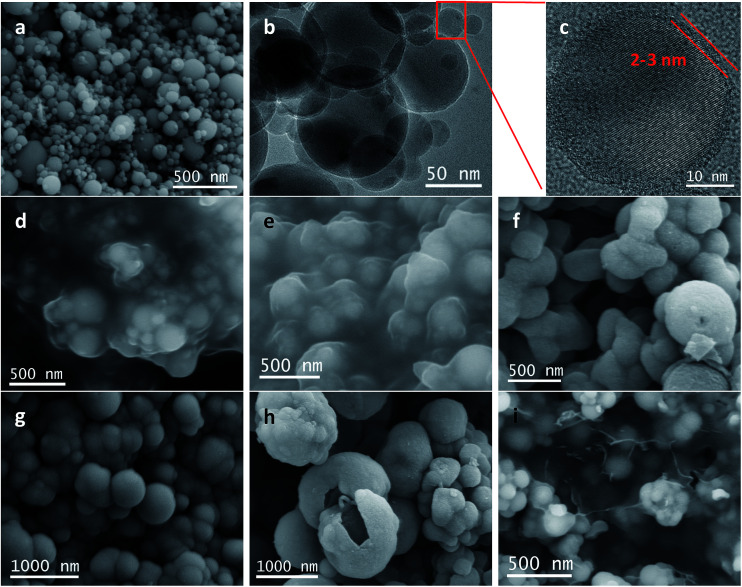
(a) SEM, (b) TEM, (c) magnified TEM images of Si nanoparticles, and corresponding SEM images of (d) Si@RF-1, (e) Si@RF, (f) Si@C-1@SiO_2_, (g) Si@C@SiO_2_, (h) Si@void@SiO_2_ and (i) Si@C@SiO_2_/RGO.

The schematic flowchart in Fig. 1 shows the main steps in the process used in this study. First, commercial silicon nanoparticles with size ranging from 40 to 250 nm (Fig. 2a and b) were coated with a critically designed RF layer prepared *via* a sol–gel process (Fig. 2d and e). A homogeneous RF layer and the inner silicon core could be easily observed through SEM. The magnified TEM image shows that there is a thin layer of silica (2–3 nm) adhered on the surfaces of silicon nanoparticles, which improves the dispersion of silicon in aqueous solution to conduct the subsequent processes (Fig. 2c). Moreover, the thin silicon oxides layer could ensure a negatively charged interface, promoting CTAB adhesion to the surfaces and making the self-assembly of RF easier.^32^ Subsequently, the SiO_2_ layer was coated by the commonly used Stöber method in a mixed solution of deionized water and ethanol. Part of the obtained Si@RF@SiO_2_ particles were calcined in argon atmosphere and the RF layer was transformed into a carbon layer. All the samples exhibit a smooth spherical surface (Fig. 2f and g). In addition, the carbon layer, as observed from the ruptured area, is closely linked with the core and shell due to the addition of CTAB, which is consistent with the previous report.^33^ The other part of the samples were calcined in air, following which the RF layer was oxidized to form a cavity between the SiO_2_ shell and the Si core. Due to the escaping of the gas caused by RF layer decomposition, a small portion of the shells broke down (Fig. 2h). It is worth mentioning that the cavity size of the sample is similar to the carbon layer thickness of the carbonized sample because the cavity is directly generated by *in situ* removal of RF. Thus, the two structures had experimental comparability. In case of the Si@C@SiO_2_/RGO composites, the Si@C@SiO_2_ particles were uniformly dispersed between thin RGO layers (Fig. 2i). The wrinkled graphene sheets could further improve the conductivity and promote the cycling performance.^34,35^

In the double core–shell structure, the two layers have their own unique advantages. First, the SiO_2_ layer is prepared by Stöber method. (1) It is strong enough to limit the expansion of silicon nanoparticles and maintain the structural stability with minimal volume change during cycling. (2) It has good thermal stability, so that the structural collapse is avoided during the high temperature carbonization. (3) It has better ion transport ability than silicon, which ensures a preeminent electrochemical performance. (4) During cycling, its negligible volume change ensures the formation of thin and stable SEI layer on the electrode surfaces.^36,37^ Second, the resorcinol–formaldehyde resin (RF) layer, fixed by silica layer and silicon core along with the addition of cetyl trimethylammonium bromide (CTAB) as template agent, is transformed into intermediate carbon layer due to high temperature carbonization. CTAB can not only generate pores to provide channels for ion transport, but also prevent the shrinkage of carbon layer on one side (core or outer shell) during carbonization.^33^ The synergistic effects of CTAB and shrinking of RF are beneficial in the fabrication of a loose and highly porous carbon layer. Such rationally designed carbon shell has multiple advantages: (1) it is loose enough and has a certain elasticity, both of which can alleviate the expansion of silicon during cycling;^38^ (2) these porous structures provide channels for ion transport without much initial specific discharge capacity loss;^39^ (3) the carbon layer is in close contact, named face-to-face contact, with silica shell and silicon core, which ensures better electron/ion transport than the yolk–shell structure (point-to-point contact).

**Fig. 3:**
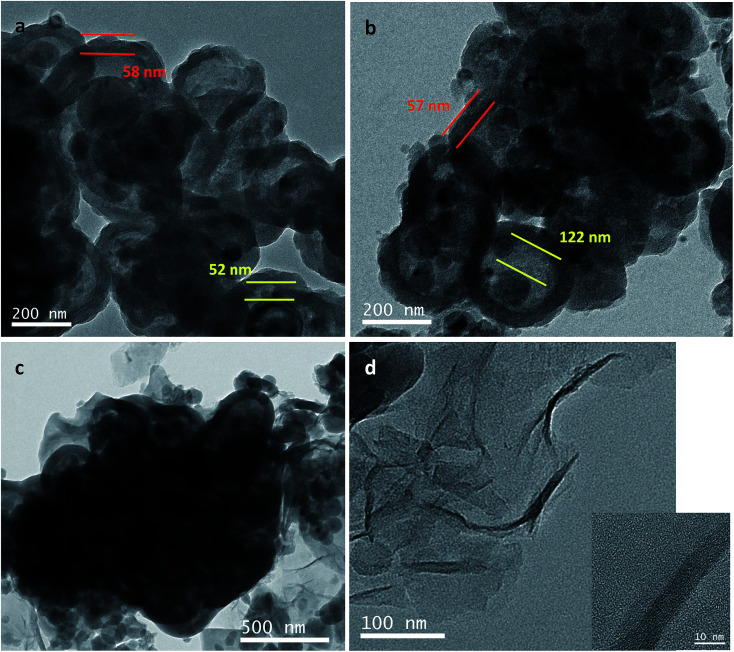
TEM images of (a) Si@C@SiO_2_, (b) Si@void@SiO_2_, (c) Si@C@SiO_2_/RGO and (d) TEM of RGO separated from Si@C@SiO_2_/RGO (inset is high magnification image).

TEM images of Si@C@SiO_2_, Si@void@SiO_2_, and Si@C@SiO_2_/RGO are shown in Fig. 3. In case of the Si@C@SiO_2_ sample, due to the existence of the intermediate carbon layer produced by the *in situ* carbonization of RF, a pattern of concentric circles is observed in the TEM image as the silicon particles are in the middle of the spheres (Fig. 3a). However, after the oxidation of the RF layer, the voids were formed in the middle of the structure. Silicon particles were no longer fixated by carbon in the spheres' centres, but were eccentric (Fig. 3b). The SiO_2_ layers of the two samples had the same thickness (52 ± 6 nm, red line in Fig. 3a and b), indicating that high temperature treatment did not have a negative impact on SiO_2_. At the same time, the thickness of carbon layer in Si@C@SiO_2_ is approximately equal to half the size of cavity in Si@void@SiO_2_, both of which are 55 ± 5 nm (yellow line). Fig. 3c represents the TEM image of Si@C@SiO_2_/RGO. It could be observed that Si@C@SiO_2_ maintains the original shape and surrounded by RGO layers, indicating the homogeneous mixing of the two composites. Fig. 3d shows a piece of isolated RGO with a uniform distribution and the inset figure is its high magnification TEM, from which the layered structure of RGO is clearly observed.

**Fig. 4:**
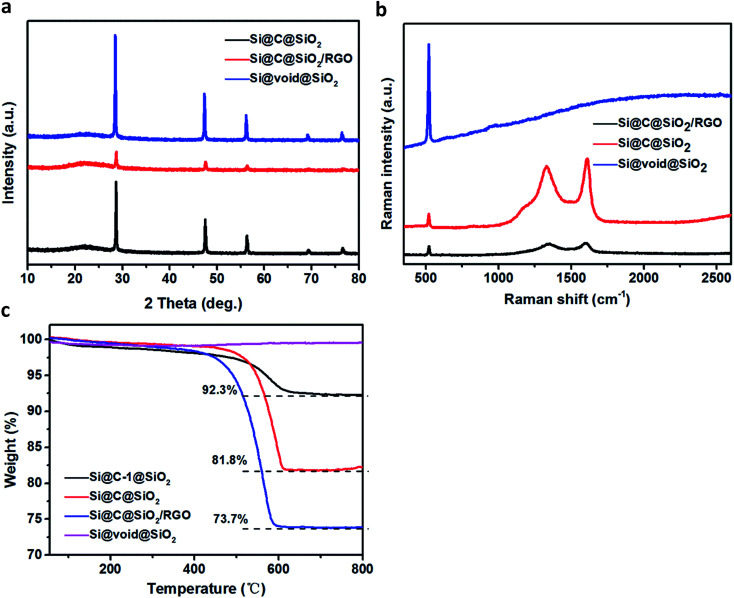
(a) XRD patterns and (b) Raman spectra of Si@C@SiO_2_, Si@C@SiO_2_/RGO and Si@void@SiO_2_, (c) TGA curves of Si@C-1@SiO_2_, Si@C@SiO_2_, Si@C@SiO_2_/RGO and Si@void@SiO_2_.

Fig. 4a shows the XRD pattern of the three samples: Si@C@SiO_2_, Si@C@SiO_2_/RGO, and Si@void@SiO_2_. The XRD pattern of each sample displays five sharp peaks at 2*θ* value of 28.6°, 47.5°, 56.3°, 69.1° and 76.6°, corresponding to (111), (220), (311), (400), and (331) crystalline faces of silicon, respectively. The pattern for Si@void@SiO_2_ sample displays a broad peak near 2*θ* ∼ 22°, due to the existence of amorphous silica.^40^ After amorphous carbon was filled in the cavity (Si@C@SiO_2_), the intensity of the amorphous broad peak increased, while the relative peak intensity of crystalline silicon peaks decreased.^41^ No peak of graphene oxide (2*θ* ∼ 10.1°) is found in the pattern of Si@C@SiO_2_/RGO, confirming that graphene oxide was completely reduced to reduced graphene oxide.^42^ After Si@C@SiO_2_ combined with RGO, the peak intensity of silicon peaks decreased sharply and the peak at 2*θ* ∼ 22° broadened. Attributed to the introduction of RGO, an ordered peak of carbon near 2*θ* ∼ 25° (002) is well-indexed.^43,44^ To gain further information regarding the structure of these samples, Raman spectra were recorded (Fig. 4b). There are strong peaks at around 522 cm^−1^ in the spectra of the three samples; these peaks were ascribed to Si.^45^ The two peaks located at around 1340 and 1605 cm^−1^ are ascribed to the D and G bands, respectively.^46^ These peaks were not observed in the spectra of Si@void@SiO_2_, indicating the complete disappearance of carbon. Compared to that of Si@C@SiO_2_, the spectra of Si@C@SiO_2_/RGO showed a blue shift of D peak from 1330 to 1346 cm^−1^ and a red shift of G peak from 1609 to 1602 cm^−1^. The shifts of the two peaks indicated that the addition of RGO improved the ordering of carbon atoms.^47^ Complete disappearance of RF layer during the transformation from Si@C@SiO_2_ into Si@void@SiO_2_ was also synergistically proved by thermogravimetric analysis (TGA, Fig. 4c), from which it is observed that the curve of Si@void@SiO_2_ rose slightly instead of declining. The other three samples containing carbon displayed weight loss between 400 and 650 °C because of the oxidation of carbon. Carbon contents of Si@C-1@SiO_2_ and Si@C@SiO_2_ were 92.3% and 81.8%, respectively. From the weight losses of Si@C@SiO_2_ and Si@C@SiO_2_/RGO, RGO content of the latter could be calculated to be 8.1%.

**Fig. 5:**
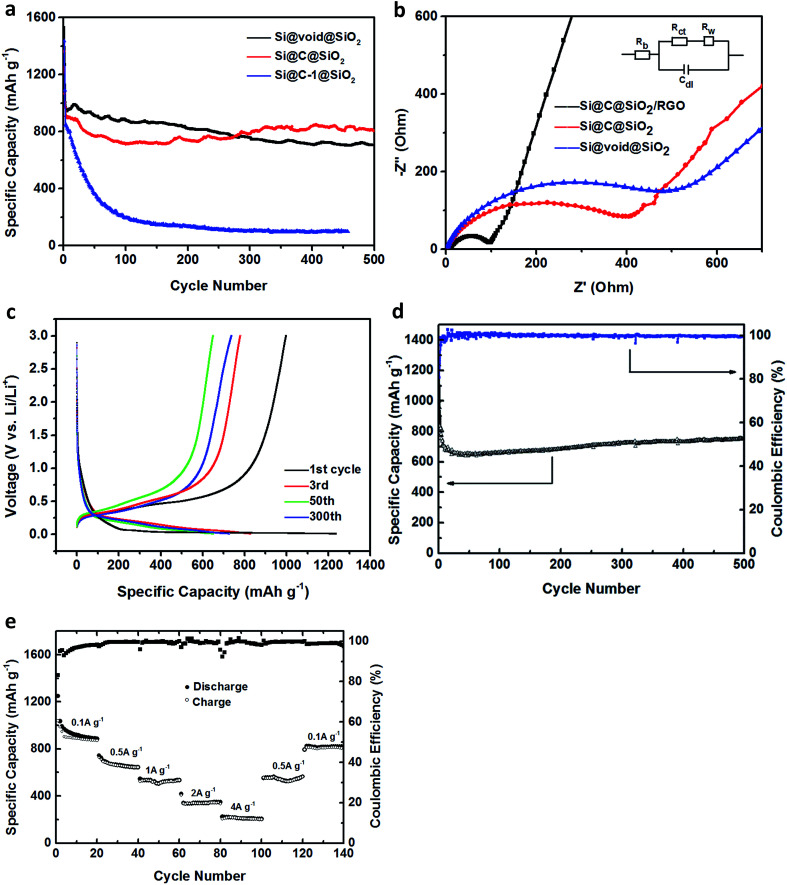
(a) The cycling performance of Si@C@SiO_2_, Si@C-1@SiO_2_, Si@void@SiO_2_ for 500 cycles between 0.01 and 3.0 V at a current density of 100 mA g^−1^ for first cycle and followed with 500 mA g^−1^, (b) the electrochemical impedance plots of Si@C@SiO_2_, Si@void@SiO_2_ and Si@C@SiO_2_/RGO, (c) the discharge–charge curves, (d) the cycling performance and the coulombic efficiency, and (e) the rate performance of Si@C@SiO_2_/RGO.

To compare the electrochemical performance, these nanocomposites were made into working electrodes and the lithium foil was employed as the counter electrode to test the button cells. Fig. 5a shows the specific capacities of Si@C-1@SiO_2_ and Si@void@SiO_2_ at the charging/discharging current density of 100 mA g^−1^ in the first two cycles and then at a current density of 500 mA g^−1^. Because the silicon content of Si@void@SiO_2_ material was higher and had a certain cavity for the expansion/contraction of silicon, its initial specific capacity and stability of the first 100 cycles were superior. However, after the 276^th^ cycle, it was surpassed by Si@C@SiO_2_ as its performance increased gradually after the 150^th^ cycle. The earlier decline was due to the presence of large number of defects in the intermediate carbon shell, which resulted in an irreversible storage of lithium ions and formation of SEI layer.^39^ The recovery after the 150^th^ cycle might be due to the gradual activation of the electrode during the cycling process, which promoted the abjection of lithium ions and the reversible formation/dissolution of the SEI layer. This phenomenon was also observed in other anodes.^48,49^ For Si@C-1@SiO_2_, the specific capacity reduced to approximately zero because this material was capable of buffering the volume expansion of silicon during cycling and thus destroyed the structure. It is worth mentioning that the thickness of the carbon layer could guarantee a stable cycling performance without the limitation of the outside silica layer. However, in this structure, structural stability could not be maintained because of the limitation of silica layer, which made the carbon layer unable to exert its elasticity to a greater extent.

The electrochemical impedance spectroscopy (EIS) of Si@C@SiO_2_ and Si@void@SiO_2_ was tested before cycling (Fig. 5b) to study why the two structures showed different results in the battery performance. The test spectrum had two parts: the semicircle at high frequency and the oblique line at low frequency. The small graph in the inset represents the equivalent circuit model of EIS, in which *R*_b_, the initial resistance, reflected the conductivity of the electrodes, electrolyte and separator and was determined from the value of intersection of the spectrum line and abscissa. *R*_ct_ and *C*_dl_ are the charge transfer resistance and the double layer capacitance, respectively, which are determined from the trend of semicircle in the spectrum. Furthermore, *R*_w_ is the lithium-ion diffusion resistance in battery, which is closely related to the linear slope at low frequency in the spectrum.^50^ In Fig. 5b, the red curve and the blue curve corresponded to the EIS plots of Si@C@SiO_2_ and Si@void@SiO_2_, respectively, and the two curves display almost the same *R*_b_ and *R*_w_ values, indicating that they had the same internal resistance and capacity of lithium-ion diffusion; the difference was that the semicircles values were 400 and 498 Ω, respectively. This indicated that Si@C@SiO_2_ had excellent charge diffusion ability and this advantage might promote the activation of active materials, thus affecting the performance of the battery.

There is no doubt that yolk–shell structure has more advantages:^51^ (1) it provides a suitable void for the volume expansion of silicon to protect the structural stability; (2) it avoids the contact between silicon and electrolyte with stable SEI layer and reduction in electrolyte loss. However, at the beginning of lithiation, the contact area of silicon core and shell material is just one point,^52,53^ which severely limits the electron/ion transport efficiency. Although core–shell structure is not as good as the yolk–shell structure with regards to the structural stability, the shell might contact the core closely during the entire cycling process. As a result, the lithium-ion transport is not restricted (Fig. S1†). Therefore, by using suitable materials, the shell can effectively tolerate the expansion of silicon without rupture, thus improving the cycling performance.

To understand the mechanism of the superior performance of the well-designed Si@C@SiO_2_, the possible structure evolutions of Si@C@SiO_2_ and Si@C-1@SiO_2_ during cycling are shown in Fig. S3.† In case of Si@C-1@SiO_2_, the thinner carbon layer could not tolerate the unexpected volume change of silicon core. After cycling, the two shells gradually cracked and fractured, causing increasing electrolyte decomposition and SEI layer deterioration. SEM images were recorded before and after 23 cycles to prove this mechanism (Fig. S2†). A considerable amount of distinct cracks could be observed in the surface of Si@C-1@SiO_2_ (Fig. S2c†) and a noticeable thickness expansion (30.8%) is observed in the cross-section image (Fig. S2d†), indicating the volume expansion of the silicon particles, instability of this structure with a thin carbon layer as well as high stress created by the SEI formation. In contrast, Si@C@SiO_2_ showed uniform films and minor thickness expansion (12.6%) (Fig. S2e and f†). Furthermore, the homogeneous spheroidal particles after deep cycling of Si@C@SiO_2_ are observed in the inset image (Fig. S2e†) with similar shape before cycling (Fig. S2f†). However, any uniform or distinct deep cycled particles could not be found in the top-view images of Si@C-1@SiO_2_. This might be because Si@C@SiO_2_, with a suitable carbon layer, had more stable structure as well as SEI layer as compared to Si@C-1@SiO_2_.

To further improve its charge diffusion and lithium-ion diffusion abilities, RGO and Si@C@SiO_2_ were combined. Fig. 5c shows the discharge–charge curves of Si@C@SiO_2_/RGO composite anode between 0.01 and 3.0 V of the first, third, fiftieth and three-hundredth cycles. In the first discharge cycling, there was a sustained voltage drop between 1.1–0.4 V, which described the formation of SEI layer on the compound surface due to the reduction and deposition of electrolyte.^54^ This trend was not observed in the subsequent cycles, indicating that the structure remained intact without rupture, which prevented the reformation of SEI.^55^ In contrast, there was a short plateau between 0.4 V and 0.1 V and a distinct plateau profile at 0.1 V, which reflected the insertion of lithium ions into silicon/silica and carbon, respectively. In addition, voltage plateaus between 0.3 V and 0.5 V and the other plateau located at 0.8 V were well maintained during all the charge cycling processes. The two plateaus represent the extraction of lithium ions from lithiated silicon and silica.^56,57^ At 0.1 A g^−1^ current density in the first cycle, the charge and discharge capacities were 997.8 and 1236 mA h g^−1^, respectively, with an initial coulombic efficiency of 80.7%. The superior coulombic efficiency should contribute to the even dispersion of Si@C@SiO_2_ particles between RGO layers, which reduced the contact area between the electrode material and electrolyte. The irreversible capacity loss of Si@C@SiO_2_/RGO anode in the first cycle was because of the formation of SEI layer on the electrode surface. The third, fiftieth and three-hundredth cycles were carried out under a current density of 0.5 A g^−1^. The specific discharge capacities of the third and fiftieth cycles were 828.6 and 647.7 mA h g^−1^, respectively. However, the discharge capacity increased to 723.2 mA h g^−1^ at the 300^th^ cycle. This phenomenon might be due to the addition of reduced graphene oxide that could have improved charge diffusion and lithium-ion diffusion abilities of the electrode (Fig. 5b).

Fig. 5d shows the cycling performance of Si@C@SiO_2_/RGO and the coulombic efficiency. The first discharge specific capacity reached 1263 mA h g^−1^, but decreased to 662 mA h g^−1^ at the 21^st^ cycle due to the polarization of the electrode and the formation of SEI. Then, it slowly returned to 753.8 mA h g^−1^ at the 500^th^ cycle, which was 91% of the value of the third cycle (828.6 mA h g^−1^), *i.e.*, the first cycle carried out at 0.5 A g^−1^ current density. The coulombic efficiency at the 500^th^ cycle was maintained at more than 99.5%. Because of the electrode particles purify during charging and discharging^58^ and the addition of RGO, the conductivity and lithium ions transport capacity of the electrode improved, such that the specific capacity constantly rose until the 500^th^ cycle.^59^ This phenomenon indicated the switching of lithium ions insertion and extraction reactions after deep cycling, leading to the activation of active substances to varying degrees.^60^Fig. 5e represents the rate performance of the electrode at different current densities. The first 20 cycles were carried out under the current density of 0.1 A g^−1^ with the discharge specific capacity of 886 mA h g^−1^ at the 20^th^ cycle. The next 20 cycles were carried out at 0.5, 1, 2 and 4 A g^−1^ and the final capacity at different periods were 644.6, 534.2, 346.7 and 205 mA h g^−1^, respectively. These values were 75%, 60.3%, 39.1% and 23.1% of the 20^th^ cycle respectively. When the current density returned from 4 A g^−1^ to 0.5 and 0.1 A g^−1^, 87.7% and 92.5% of the specific capacities still remained at the same current densities although it could not be fully returned to the previous value. According to above mentioned results, the Si@C@SiO_2_/RGO electrode not only showed an impressive cycling performance, but also a high rate capability.

Table S1† summarises the characteristics of Si with diverse structures composited with graphene, which was prepared by various techniques, comprising the preparation method, graphene content of the composite and their electrochemical performance as anode material for lithium-ion batteries.

## 4 Conclusion

In brief, to explore the effects of the contact area between core and shell on electrochemical performance, yolk–shell structure and double core–shell structure were designed by similar processes. In contrast, at a certain thickness (around 55 nm) of carbon layer (Si@C@SiO_2_), the carbon buffer layer might effectively alleviate the damage of silicon volume expansion. The results showed that, at the same charge and discharge current density, its specific capacity could exceed that of Si@void@SiO_2_ after the 276^th^ cycle because of the increase in electron/ion transport ability of Si@C@SiO_2_, resulting from face-to-face contact. Moreover, we further improved the conductivity of materials by combining them with RGO layers and as a result, the electrochemical performance of these composites could be further improved.

## Conflicts of interest

We declare that we do not have any commercial or associative interest that represents a conflict of interest in connection with the work submitted.

## Supplementary Material

RA-008-C7RA13606D-s001
