# Supplementary material for: Fabrication of double core–shell Si-based anode materials with nanostructure for lithium-ion battery
Source: RSC Adv. 2018 Mar 1;8(17):9094–102. doi: 10.1039/c7ra13606d (PMC9078599; doi:10.1039/c7ra13606d)
Supplement: RA-008-C7RA13606D-s001 [file RA-008-C7RA13606D-s001.pdf]

Electronic Supplementary Material (ESI) for RSC Advances.
This journal is © The Royal Society of Chemistry 2018

**Supplementary information**

**Figure S1.** Pathways of lithium-ion transport in two different structures

**Figure S2.** SEM images of the anode before and after the cycling tests for 23 cycles, in which the current density cycle is 100 mA $g^{-1}$ for the first three cycles and 500 mA $g^{-1}$ for the followed 20 cycles. The top-view images were shown in the left while the cross-section was laid in the right. a) and b) anode befor cycling, c) and d) Si@C-1@$SiO_2$ after cycling. e) and f) Si@C@$SiO_2$ after cycling.

**Figure S3.** Schematic illustration of the possible structure evolutions of double core-shell Si@C-1@$SiO_2$ and Si@C@$SiO_2$ during cycling

**Table S1.** The comparision of the performance and preparation method between this paper and previous work

**Figure S1**

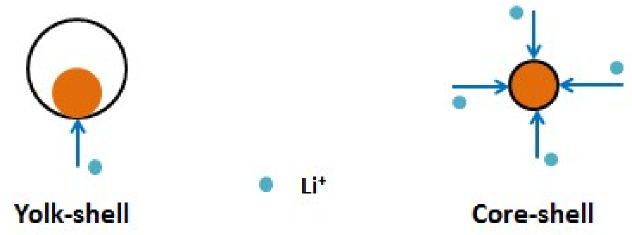

**Figure S2**

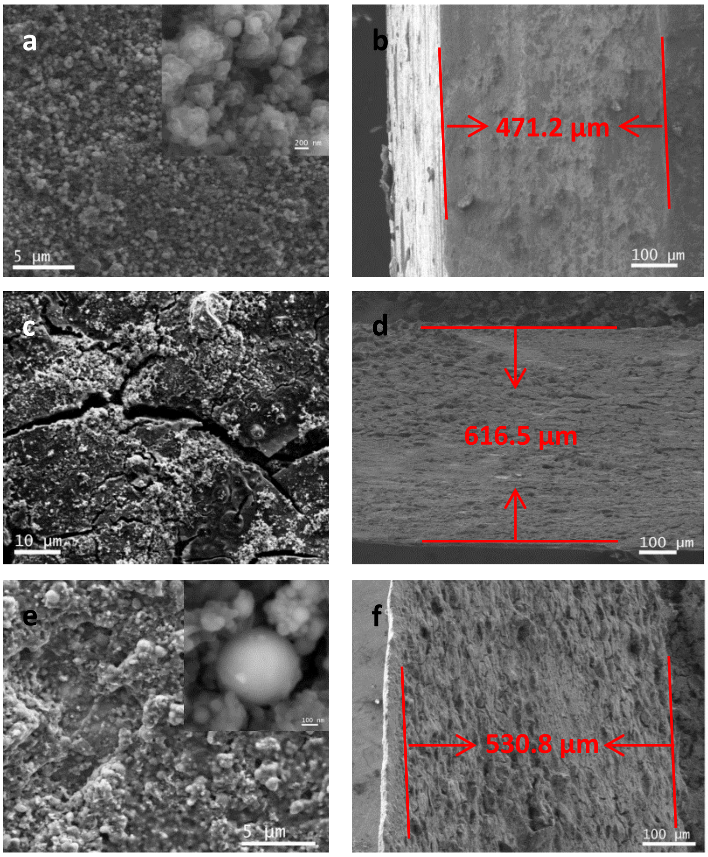
a
200 nm
5 µm
b
471.2 µm
100 µm
c
10 µm
d
616.5 µm
100 µm
e
100 nm
5 µm
f
530.8 µm
100 µm

**Figure S3**

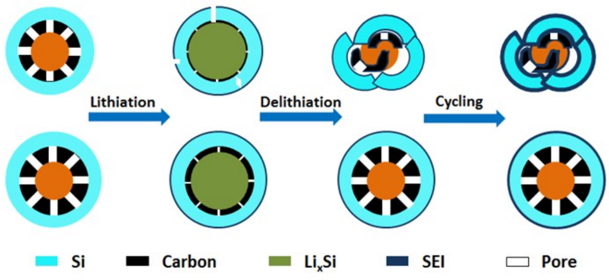

Table S1

| Anode materials | Graphene preparation method | Graphene content (wt%) | Initial CE | Initial capacity | Capacity remained | Current density | Ref. |
|---|---|---|---|---|---|---|---|
| **C coated Si microparticles/RGO** | GO Ar/$H_2$ reduced | ~9.7% | 91.1% | 1947 mAh $g^{-1}$ at 200 mA $g^{-1}$ | 73% after 157 cycles | 200 mA $g^{-1}$ | 1 |
| **C coated Si nanoparticles/RGO** | GO Ar/$H_2$ reduced | _ | ~71% | 4209 mAh $g^{-1}$ at 200 mA $g^{-1}$ | ~38.1% after 350 cycles | 2 A $g^{-1}$ | 2 |
| **Si nanoparticles/ nanographene** | CVD graphene growth | ~8% | 83.4% | 2330 mAh $g^{-1}$ at 250 mA $g^{-1}$ | 95% after 510 cycles | 500 mA $g^{-1}$ | 3 |
| **Si nanoparticles/RGO** | GO Ar/$H_2$ reduced | ~60% | 83% | 645 mAh $g^{-1}$ at 50 mA $g^{-1}$ | 98.5% after 100 cycles | 50 mA $g^{-1}$ | 4 |
| **2D Ag coated Si nanoparticles/RGO** | GO $N_2$ reduced | ~10% | 70% | 2419 mAh $g^{-1}$ at 100 mA $g^{-1}$ | 70.2% after 100 cycles | 1 A $g^{-1}$ | 5 |
| **Microsized porous silicon/graphenen** | GO aluminum reduced | ~80% | 80.96% | ~3150 mAh $g^{-1}$ at 1000 mA $g^{-1}$ | 39.9% after 300 cycles | 1 A $g^{-1}$ | 6 |
| **Yolk-shell Si@RGO/a-c** | GO Ar reduced | ~6% | 76 % | ~2200 mAh $g^{-1}$ at 400 mA $g^{-1}$ | 75% after 101cycles | 400 mA $g^{-1}$ | 7 |
| **Double core-shell Si nanoparticles/RGO** | GO Ar/$H_2$ reduced | ~8.1% | 80.7% | 1263 mAh $g^{-1}$ at 100 mA $g^{-1}$ | 91% after 500 cycles | 500 mA $g^{-1}$ | This work |

1. X. Han, H. Chen, Z. Zhang, D. Huang, J. Xu, C. Li, S. Chen and Y. Yang, *Journal of Materials Chemistry A*, 2016, **4**, 17757-17763.
2. Z. Liu, P. Guo, B. Liu, W. Xie, D. Liu and D. He, *Applied Surface Science*, 2017, **396**, 41-47.
3. C. Zhang, T.-H. Kang and J.-S. Yu, *Nano Research*, 2018, **11**, 233-245.
4. C. Botas, D. Carriazo, W. Zhang, T. Rojo and G. Singh, *ACS Applied Materials & Interfaces*, 2016, **8**, 28800-28808.
5. W. Yao, Y. Cui, L. Zhan, F. Chen, Y. Zhang, Y. Wang and Y. Song, *Applied Surface Science*, 2017, **425**, 614-621.
6. W. Zhai, Q. Ai, L. Chen, S. Wei, D. Li, L. Zhang, P. Si, J. Feng and L. Ci, *Nano Research*, 2017, **10**, 4274-4283.
7. M.-H. Lin, S. Hy, C.-Y. Chen, J.-H. Cheng, J. Rick, N.-W. Pu, W.-N. Su, Y.-C. Lee and B.-J. Hwang, *ChemElectroChem*, 2016, **3**, 1446-1454.
